# Contrastive self-supervised learning for neurodegenerative disorder classification

**DOI:** 10.3389/fninf.2025.1527582

**Published:** 2025-02-17

**Authors:** Vadym Gryshchuk, Devesh Singh, Stefan Teipel, Martin Dyrba, the ADNI, AIBL, FTLDNI study groups

**Affiliations:** ^1^German Center for Neurodegenerative Diseases (DZNE), Rostock, Germany; ^2^Department of Psychosomatic Medicine, Rostock University Medical Center, Rostock, Germany

**Keywords:** contrastive learning, self-supervised learning, neurodegenerative disorders, deep learning, structural magnetic resonance imaging, Alzheimer's disease, frontotemporal lobar degeneration

## Abstract

**Introduction:**

Neurodegenerative diseases such as Alzheimer's disease (AD) or frontotemporal lobar degeneration (FTLD) involve specific loss of brain volume, detectable *in vivo* using T1-weighted MRI scans. Supervised machine learning approaches classifying neurodegenerative diseases require diagnostic-labels for each sample. However, it can be difficult to obtain expert labels for a large amount of data. Self-supervised learning (SSL) offers an alternative for training machine learning models without data-labels.

**Methods:**

We investigated if the SSL models can be applied to distinguish between different neurodegenerative disorders in an interpretable manner. Our method comprises a feature extractor and a downstream classification head. A deep convolutional neural network, trained with a contrastive loss, serves as the feature extractor that learns latent representations. The classification head is a single-layer perceptron that is trained to perform diagnostic group separation. We used *N* = 2,694 T1-weighted MRI scans from four data cohorts: two ADNI datasets, AIBL and FTLDNI, including cognitively normal controls (CN), cases with prodromal and clinical AD, as well as FTLD cases differentiated into its phenotypes.

**Results:**

Our results showed that the feature extractor trained in a self-supervised way provides generalizable and robust representations for the downstream classification. For AD vs. CN, our model achieves 82% balanced accuracy on the test subset and 80% on an independent holdout dataset. Similarly, the Behavioral variant of frontotemporal dementia (BV) vs. CN model attains an 88% balanced accuracy on the test subset. The average feature attribution heatmaps obtained by the Integrated Gradient method highlighted hallmark regions, i.e., temporal gray matter atrophy for AD, and insular atrophy for BV.

**Conclusion:**

Our models perform comparably to state-of-the-art supervised deep learning approaches. This suggests that the SSL methodology can successfully make use of unannotated neuroimaging datasets as training data while remaining robust and interpretable.

## 1 Introduction

Neurodegenerative diseases such as Alzheimer's disease (AD) and frontotemporal dementia (FTD) are characterized by specific brain volume loss, which can be assessed *in-vivo* using structural magnetic resonance imaging (MRI). The usual radiological evaluation of MRI scans is performed mainly by visual examination, which is often time-consuming. Assistance systems for the automated detection of disease-specific patterns could be useful for better clinical diagnosis, as they can significantly decrease the evaluation time for radiologists and neurologists, and help them focus on relevant brain regions. Convolutional neural networks (CNNs) models can automatically identify neurodegenerative diseases from MRI scans and achieve state-of-the-art results in medical imaging tasks. Recent developments in the CNN architectures have in turn shaped the neuroimaging community, which is interested in automatic discovery of image features pertinent to neurological illnesses. Various tasks, such as disease diagnosis, pathology localization, anatomical region segmentation, etc., now rely on the use of CNNs (Dyrba et al., 2021; Qiu et al., 2020; Eitel et al., 2021; Wen et al., 2020; Han et al., 2022). CNN models are primarily trained in a *supervised* manner by using an external ground-truth label. Generating such labels for data samples is often burdensome and costly. Furthermore, CNN models require a large amount of training data to achieve competitive results. Such large datasets are not easily available within the medical domain due to the high cost of data collection and the rarity of experts for annotations.

These constraints led us to reconsider the training of CNN models in a *supervised* manner, and to explore *self-supervised learning (SSL)* approaches. The SSL methods learn without any sample labels by utilizing the internal structure of the data to generate representative features. Architectures trained in a self-supervised manner are biologically plausible, provide extensive feature space, and can compete with supervised approaches (Orhan et al., 2020).

Moreover, post hoc explanation methods have been developed within the field of eXplainable Artificial Intelligence (XAI) to interpret how deep neural networks make decisions. The XAI methods for explaining CNN models rely on local feature attribution methods, which assign a relevance score to input regions for a given input, model, and resulting output. However, only a handful of studies have explored attribution-based XAI methods within the field of self-supervised learning (SSL) applications, e.g., in the medical imaging domain (Chen et al., 2023).

The main goal of our study was to explore, in a proof-of-concept study, SSL method's ability to learn generalizable features for dementia stage and type detection from structural MRI data. We hypothesized that SSL methods could learn meaningful structural representations, and resulting models could have comparable performances to supervised models. In this paper, we trained a CNN model with the SSL setup and then evaluated it on downstream classification tasks, binary and multi-class. We also explored a saliency mapping technique for highlighting relevant input regions. The main research questions were defined as: *How does the contrastive SSL paradigm compare to the supervised learning paradigm in terms of predictive power? Are the models trained in contrastive self-supervised way on neuroimaging data interpretable?*

## 2 Background

### 2.1 Self-supervised learning

Self-supervised learning (SSL) methods learn generalizable features without any data labels or ground truth information by solving an initial auxiliary task. The pretrained SSL models are then used for specific downstream tasks, e.g., identification of neurodegenerative disorders. Models trained under the SSL approach have found application in different domains, that is, image processing (Jing and Tian, 2020), video processing (Schiappa et al., 2023), and audio processing (Liu et al., 2022a). Within the imaging domain, multiple auxiliary or so-called “pretext” tasks have been suggested previously: identifying data augmentations (Reed et al., 2021; Chen et al., 2020), rotation prediction (Chen et al., 2019), patch position prediction (Doersch et al., 2015; Noroozi and Favaro, 2016; Wei et al., 2019), image colorization (Larsson et al., 2017, 2016), and contrastive learning (Jaiswal et al., 2020).

SSL methods could be thought of as an alternative to pre-training or automated feature learning step and are related to the way how young children learn (Orhan et al., 2020). Particularly, contrastive SSL methods try to learn the general structure present within the data, by using *supervisory signals* extracted from the data itself independently of the ground truth for any specific use-case. In our study, we used contrastive learning due to its widespread application as a pretext task (Shurrab and Duwairi, 2022; VanBerlo et al., 2024).

#### 2.1.1 Formal definition of contrastive SSL

Contrastive learning tasks have received considerable attention within the SSL methods. Contrastive learning tasks aim to learn a latent space in which embeddings of similar data samples are pulled together, and embeddings of dissimilar data samples are pushed apart (Gutmann and Hyvärinen, 2010; Weng, 2021; Chopra et al., 2005). Various loss functions have been suggested to increase the quality of learned embeddings, and expedite the training. These include contrastive loss (Gutmann and Hyvärinen, 2010), triplet loss (Chechik et al., 2010; Schroff et al., 2015), N-pair loss (Sohn, 2016), InfoNCE loss (Oord et al., 2019), and Neighborhood-based loss (Sabokrou et al., 2019) etc. Contrastive learning is based on the use of positive and negative data pairs (Grill et al., 2020; Chen et al., 2020), where a *positive pair* (*i, j*) consists of two similar data instances or views. In many studies, a data sample is paired with its own augmented variations to create such positive pairs. A *negative pair* generally contains two different data samples. The contrastive loss ℓ for a positive pair is formally defined as follows.


(1) $$\begin{array}{l} \begin{array}{c} \ell \left(i,j\right)=-log\frac{exp\left(cos\left({\text{z}}_{i},{\text{z}}_{j}\right)/\tau \right)}{\sum_{k=1}^{2N}{𝟙}_{\left[k\neq i\right]}exp\left(cos\left({\text{z}}_{i},{\text{z}}_{k}\right)/\tau \right)}, \end{array} \end{array}$$

Where τ is a scaling factor called temperature, 𝟙 is an indicator function with output values being 0 or 1, *N* is the number of training samples, exp(·) is the exponential function, and cos(·) is the cosine similarity function, over different *z* latent representation of the input.

The Nearest-Neighbor Contrastive Learning (NNCLR) method (Dwibedi et al., 2021) extends the common contrastive loss by keeping a record of recent embeddings of augmented views in a queue *Q*. Thus, the pairs are not directly compared, rather a projection embedding that is most similar to a view is selected from *Q* for the comparison with another view. The NNCLR contrastive loss ℓ_*n*_ is defined as:


(2) $$\ell_{n}(i,j)=-log\frac{exp(cos(S(z_{i},Q),z_{j})/\tau )}{\sum_{k=1}^{2N}{𝟙}_{\left[k\neq i\right]}exp(cos(S(z_{i},Q),z_{k})/\tau )},$$

where *S*(**z**, *Q*) is the nearest neighbor function:


(3) $$\begin{array}{l} S(z,Q)=\underset{q\in Q}{\arg\;\min}{\left\|z-q\right\|}_{2}. \end{array}$$

#### 2.1.2 Self-supervised learning in medical imaging

Recent advancements in self-supervised learning (SSL) facilitate the training of models capable of effectively acquiring feature representations relevant to downstream tasks (Thomas et al., 2024; VanBerlo et al., 2024). When applied to imaging data, SSL methodologies primarily focus on image reconstruction (Hu et al., 2021a; Zhou et al., 2023), segmentation (Taleb et al., 2020; Sun et al., 2023), denoising (Pfaff et al., 2024), and disease classification (Dufumier et al., 2021; Jiang and Miao, 2022; Gorade et al., 2023). For example, the study by Taleb et al. (2020) introduces SSL pretext tasks, including patch-based prediction of latent representations and the augmentation prediction. In contrast, Hu et al. (2021a) suggests an alternative pretext task leveraging two parallel networks to minimize reconstruction loss. Additional research has used SSL on longitudinal Alzheimer's Disease (AD) MRI datasets to explore methods to integrate information from multiple imaging modalities (Fedorov et al., 2021) or to predict the trajectory of cognitive performance and/or cognitive decline (Ouyang et al., 2022; Zhao et al., 2021).

Contrary to the aforementioned studies, which aimed at applying SSL techniques for the learning of feature representations within broader application area, our work assesses the effectiveness of these representations acquired through SSL in differentiating neurodegenerative disorders with an emphasis on the interpretability of the models.

### 2.2 Convolutional neural network backbones

Convolutional neural networks (CNN) have been the state-of-the-art solutions for computer vision tasks for almost a decade. In the last few years, numerous approaches on the advancement of CNNs were proposed: introduction of skip connections (He et al., 2016; Huang et al., 2017), experimentation with model hyper-parameters such as kernel size (Ganjdanesh et al., 2023), normalization strategies (Ioffe and Szegedy, 2015) and activation functions (Dubey et al., 2022; Apicella et al., 2021), depthwise convolutions (Howard et al., 2017), and model's block architecture (Sandler et al., 2018).

With the introduction of attention priors, vision transformers (ViT) (Dosovitskiy et al., 2020) soon became a viable alternative to purely convolutional models, and currently represent the state-of-the-art model architecture as generic vision backbones. ViTs were inspired by the transformer models applied to language processing tasks. To the best of our knowledge, there weren't attempts of systematically comparing attention priors with convolutional priors. However, in their study Liu et al. (2022b) culminated many of the CNN advancements proposed over the years, and compared the resulting ConvNeXt model with comparable vision transformers. ConvNeXt (Liu et al., 2022b) was proposed as a purely convolutional model, which achieved favorable results on common vision benchmarks such as the ImageNet (Deng et al., 2009) and the COCO (Lin et al., 2014) datasets, sometimes even providing higher accuracy than competing ViT models. Notably, ConvNeXt achieved these results while maintaining the computational simplicity and efficiency of standard CNN models, highlighting the importance of convolutional priors for vision tasks.

### 2.3 Feature attribution

With the growing popularity of CNN models and these models becoming the off-the-shelf baselines, there has also been a growing need to understand them. Multiple studies have attempted to explain and interpret black-box CNN models. Within the domain of explainable AI (XAI), there are various methods to derive the importance of input features, i.e., the importance scores with respect to each prediction. These importance scores can be visualized by superimposing them on the input scans (Van der Velden et al., 2022). Certain preferred methods of importance scoring are Layer-wise Relevance Propagation (LRP) (Montavon et al., 2019; Kohlbrenner et al., 2020), Gradient-weighted Class Activation Mapping (Grad-CAM) (Selvaraju et al., 2020), and Integrated Gradients (IG) (Sundararajan et al., 2017). Multiple studies have been conducted mapping importance scores to input regions, particularly within the neuroscience application of dementia detection (Dyrba et al., 2021; Singh and Dyrba, 2023; Böhle et al., 2019; Leonardsen et al., 2024; Wang et al., 2023).

## 3 Methods

### 3.1 Neuroimaging datasets

We used T1-weighted brain MRI scans from publicly available neuroimaging repositories. The data scans in our study were pooled from the following data repositories: (i) the Alzheimer's Disease Neuroimaging Initiative (ADNI),^1^ study phases ADNI2 and ADNI3, (ii) the Australian Imaging, Biomarker & Lifestyle Flagship Study of Aging (AIBL),^2^ collected by the AIBL study group, and (iii) the Frontotemporal Lobar Degeneration Neuroimaging Initiative (FTLDNI).^3^ In our study, the cognitively normal (CN) scan samples were consolidated from all three data cohorts. The ADNI and AIBL data cohorts provided samples with dementia due to Alzheimer's disease (AD) and mild cognitive impairment (MCI). While, FTLDNI was the only data cohort with samples categorized into different frontotemporal lobar degeneration (FTLD) phenotypes, i.e., the behavioral variant of frontotemporal dementia (BV), the semantic variant of frontotemporal dementia (SV), and the progressive non-fluent aphasia (PNFA). Notably, the data from ADNI3, ADNI2 and FTLDNI was used for training all models, and AIBL was used as independent test dataset.

We applied the “t1-linear pipeline” of the Clinica Python library (Routier et al., 2021; Wen et al., 2020) to preprocess the raw MRI scans. The pipeline uses the N4ITK method for bias field correction and the SyN algorithm from ANTs to perform an affine registration for alignment of each scan with the Montreal Neurological Institute (MNI) reference space. However, more advanced steps such as brain extraction, tissue segmentation, and non-linear warping were not performed. Some MRI scans were excluded due to severe quality issues, i.e., the presence of imaging artifacts such as blurring or ghosting, or missing diagnostic information.

Additionally, each scan was cropped to the size of 169 × 208 × 179 voxels with 1 mm isotropic resolution. After applying preprocessing methods, our study includes 841 scans from the ADNI2, 968 scans from the ADNI3, 612 scans from AIBL and 273 scans from FTLDNI. Table 1 summarizes the sample statistics of the different data sources.

**Table 1:** Sample statistics of study data per diagnosis state.

	**CN**	**AD**	**MCI**	
**ADNI3**				
Age: μ(σ)	74 (7)	77 (8.3)	74.6 (8)	
MMSE: μ(σ)	29.4 (0.7)	20.8 (4.5)	27.9 (1.1)	
Sex: F/M	312/221	52/70	140/173	
**ADNI2**				
Age: μ(σ)	75.8 (7)	76.2(7.6)	74.6 (7.9)	
MMSE: μ(σ)	29.3 (0.7)	21.1(4.3)	27.8 (1.1)	
Sex: F/M	110/94	120/163	151/203	
**AIBL**				
Age: μ(σ)	73.5 (6.4)	75.4 (7.9)	76.6 (6.5)	
MMSE: μ(σ)	29.2 (0.8)	19.5 (5.8)	27.2 (1.3)	
Sex: F/M	239/182	51/37	41/62	
	**CN**	**BV**	**SV**	**PNFA**
**FTLDNI**				
Age: μ(σ)	64.3 (7.1)	62.1 (5.8)	62.7 (6.8)	68.9 (7.7)
MMSE: μ(σ)	29.7 (0.5)	22.6 (6.2)	22.5 (5.7)	24.9 (5.5)
Sex: F/M	72/58	23/48	14/23	19/16

CN, a cognitively normal state; AD, dementia due to Alzheimer's disease; MCI, mild cognitive impairment; BV, behavioral variant of frontotemporal dementia; SV, semantic variant of frontotemporal dementia; PNFA, progressive non-fluent aphasia; μ, mean; σ, standard deviation; MMSE, mini-mental state examination; F, female; M, male.

### 3.2 Proposed self-supervised learning pipeline

**Figure 1:**
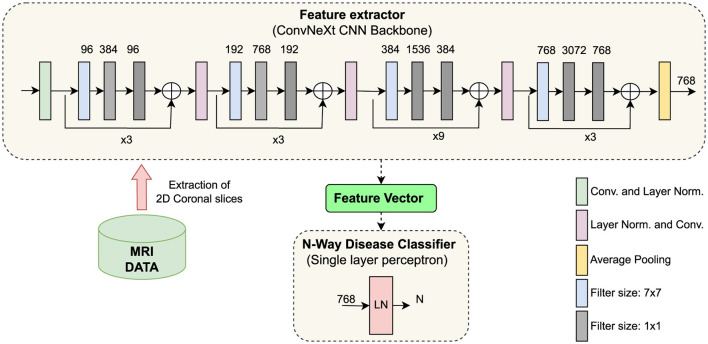
Illustration of the proposed architecture. **(Top)** ConvNeXt, a CNN model, trained under a self-supervised learning paradigm, extracts features from coronal brain slices. **(Bottom center)** The classification head learns to classify neurodegenerative disorders from the extracted features. CNN, convolutional neural network; LN, layer normalization; Conv, convolutional operation; LN, Layer Normalization.

Our proposed method consists of two modules: a feature extractor and a classification head. The feature extractor is a convolutional neural network trained without any sample labels in a self-supervised manner. The classification head is a simple neural network subsequently trained in a supervised way. The proposed architecture is shown in Figure 1.

After executing the t1-linear pipeline of the Clinica library, we obtained a 3D image for the brain of each participant. However, we only used 2D convolutional operations, as they reduce the CNN parameter space and model complexity. We selected only the coronal plane for the present study. In each MRI sample, there were in total 208 coronal slices; however, we considered only 120 coronal slices in the middle. The slices from the middle contain the relevant regions, such as the hippocampus and the temporal lobe, which are reported to be affected already in the earliest stages of Alzheimer's disease (Whitwell et al., 2008).

**Figure 2:**
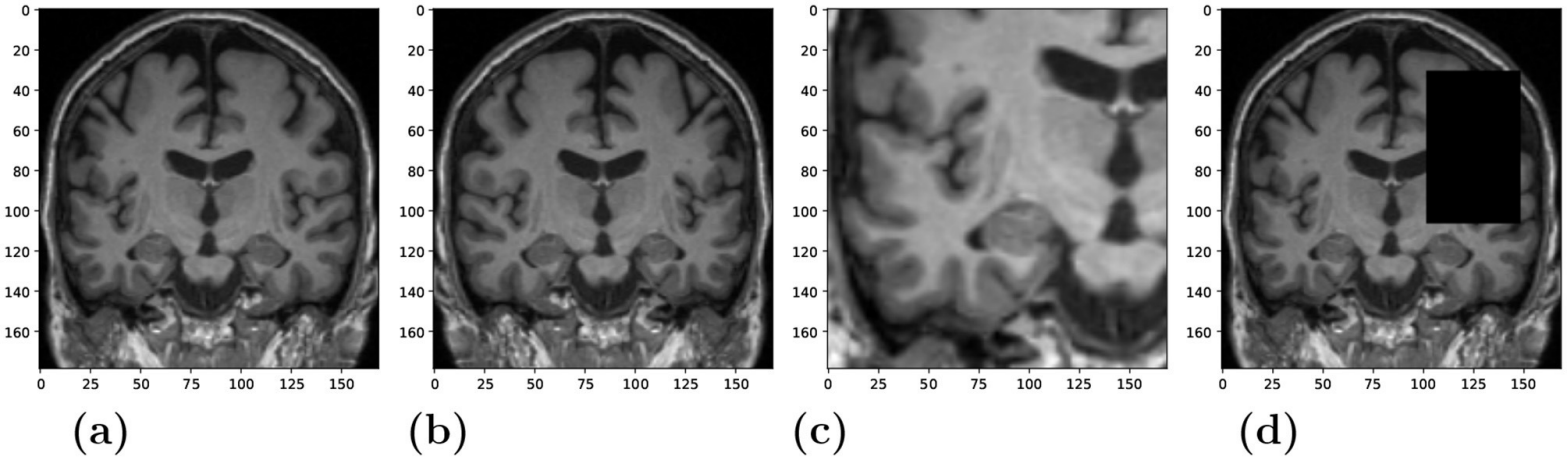
Randomly applied data augmentations to the input during training. **(A)** Original. **(B)** Horizontal flip **(C)** Crop and resize. **(D)** Occlusion.

*Feature extractor:* We used the ConvNeXt model (Liu et al., 2022b) as the backbone for the SSL framework. It was trained with the NNCLR loss ℓ_*n*_ to learn visual representations of input data (see Equation 2). We chose the NNCLR method as it provides a more generalizable learning paradigm by sampling semantic variations in the latent space and being less reliant on transformation from specific pretext tasks (Dwibedi et al., 2021). We applied a series of random augmentations to a randomly selected coronal slice for the creation of positive pairs, as exemplified in Figure 2.

The loss optimized for a data batch was:


(4) $$ℒ=\frac{1}{2N}\sum_{k=1}^{N}\left[\ell_{n}(2k-1,2k)+\ell_{n}(2k,2k-1)\right]$$

where ℓ_*n*_ is the NNCLR loss from Equation 2, 2*k* − 1 and 2*k* represent the indices of the same augmented slice, and *N* is the total number of training samples.

Specifically, we used the “tiny” variant ConvNeXt model (Liu et al., 2022b) as our backbone model. It has a configuration with sequential blocks set to (3, 3, 9, 3) and the number of output channels equalling to (96, 192, 384, 768). ConvNeXt culminates in many architectural advancements such as larger 7x7 kernel sizes, skip connections, inverted bottleneck, Gaussian error linear units (GELU) as activation function, layer-wise normalization (LN) strategy instead of batch normalisations (BN), etc. The ConvNeXt model and pretrained model weights can be downloaded from the publicly available PyTorch library (Paszke et al., 2019).

*Classification head:* While using the ConvNeXt model as a feature extractor, we considered the output produced by a 2*D* adaptive average pooling layer after the last convolutional block as input for the subsequent “classification head” (Figure 1). That means the classification head takes as input the latent feature representations of the MRI scans that where processed by the backbone CNN model. The dimension of the extracted feature vector per MRI slice is 768. Our classification head is a simple neural network consisting of a single fully-connected layer preceded by a layer normalization operation (Figure 1 bottom). A single-layer perceptron was chosen as the classification head to leverage the features extracted from the ConvNeXt feature extractor directly, and not transforming the features by applying multiple levels of nonlinearities. This design choice aims to preserve the integrity of the extracted features. Employing a single-layer perceptron is a widely recognized methodology, commonly referred to as *linear evaluation* or *linear probing* (Dubois et al., 2023; Scheibenreif et al., 2024; Kalibhat et al., 2024).

### 3.3 Feature attribution

Integrated gradients (IG) can be applied to various data modalities, such as text, images, or structured data (Sundararajan et al., 2017). IG was chosen over other feature-attribution methods because of its strong theoretical justifications, such as the completeness property of the integrated gradients. IG considers a straight path from some baseline to the input, and computes the gradients along that path. These accumulated gradients are called integrated gradients. However, this accumulation is an approximation of the actual integration of the gradients, and the number of steps taken between the baseline to the input determines the quality of this approximation. In our study, we set *N* = 50 as the number of integration steps taken between the baseline image and the input image. To calculate IG importance scores, a mean CN image was used as a baseline for the IG attribution method. We used the IG implementation provided by the Captum library (Kokhlikyan et al., 2020) to calculate importance maps for MRI scans with respect to the classification task.

### 3.4 Experimental setup

*Training the feature extractor:* We trained a feature extraction model (ConvNeXt) using the NNCLR method on ADNI3, ADNI2 and FTLDNI data for three learning trials. For each trial, we created random training and test sets. These sets were held constant for all experiments. If more than one MRI recording was available per participant, then we assigned all participant's MRI scans only to one set, thus avoiding data leakage. This resulted in 10% of data belonging to the test set.

The model was trained for 1,000 epochs using a batch size of 180 samples. The size of the NNCLR queue *Q* was set to 8,192. We applied three different data augmentation techniques with a probability of 0.5 to produce views visualized in Figures 2B–D: horizontal flip, cropping and resizing, and occlusion. We experimented with different data sources to train the feature extractor, i.e., utilizing in-domain medical images vs. training with out-of-domain natural images. More details about model training and results could be found in the supplementary.

*Training the classification head:* To determine if a 3D MRI scan belongs to a specific diagnostic group, we first derive the latent representation vectors for 2D coronal slices using the ConvNeXt feature extractor and then make a prediction for each slice using the classification head. For evaluation with the test data, we applied a majority voting procedure in which the group label that occurs the most frequently determined the final group assignment. We trained the classification head for 100 epochs, on the same three training trials that were used to train the feature extractors. We used a batch size of 64 samples and decayed the learning rate with cosine annealing after every 20 epochs.

We experimented with various setups for training a classification head while keeping the weights of the feature extractor frozen vs. unfrozen, i.e., letting the weights change during the classification head training. For the downstream task, we compared different multi-class classification heads, i.e., predicting four (CN, MCI, AD, BV) or three classes—(CN, MCI, AD) and (CN, AD, BV), and binary classification heads—(CN, AD), (CN, BV), and (AD, BV). Furthermore, we evaluated our models on the independent AIBL dataset, which was not used during training. The independent test dataset enabled us to assess the generalizability of our approach.

We used balanced accuracy, sensitivity (true positive rate), specificity (true negative rate), and the Matthews correlation coefficient (MCC) as evaluation metrics. Due to the class imbalance in our dataset, we have chosen balanced accuracy over simple accuracy in our study. Balanced accuracy is the average of the true positive rate and the true negative rate, and thus avoids the overestimation of model quality that (simple) accuracy generally shows in class imbalance scenarios. With the true positives *TP*, true negatives *TN*, false positives *FP*, and false negatives *FN*, the balanced accuracy is defined as:


(5) $$\begin{array}{l} \text{Balanced Accuracy}=\frac{\frac{\text{TP}}{\text{TP}+\text{FN}}+\frac{\text{TN}}{\text{TN}+\text{FP}}}{2} \end{array}$$

As shown in Chicco and Jurman (2020), the MCC should be preferred over the (simple) accuracy and the F1 score, as they could generate misleading results in unbalanced data sets. The MCC ranges between [−1, 1]. To achieve a high MCC score, the classifier would have to make correct predictions on both the majority and minority classes. The MCC is formally defined as:


(6) $$\begin{array}{l} \text{MCC}=\frac{\text{TP}\cdot \text{TN}-\text{FP}\cdot \text{FN}}{\sqrt{\left(\text{TP}+\text{FP})\cdot (\text{TP}+\text{FN})\cdot (\text{TN}+\text{FP})\cdot (\text{TN}+\text{FN}\right)}} \end{array}$$

## 4 Results

### 4.1 Diagnostic group separation

We evaluated the manner in which the classification head could be configured. We compared multi-class vs. binary classification heads. Table 2 shows the results achieved with our proposed architecture for the identification of neurodegenerative disorders, using a frozen ConvNeXt feature extractor trained under the NNCLR SSL paradigm on brain images. The reported numbers were averaged over three learning trials. For the binary (AD vs. CN) classification model, the balance accuracy reached 82% for the cross-validation test sets and 80% for the independent AIBL data cohort.

**Table 2:** Classification results of our proposed architecture, consisting of a frozen feature extractor trained under a SSL paradigm, and a single-layer neural network as the downstream classification head.

	**Balanced accuracy**	**MCC**	**Sensitivity**	**Specificity**
**Cross-validation test set (ADNI2/3 and FTLDNI)**				
AD vs. MCI vs. CN vs. BV:	0.60 ± 0.03	0.32 ± 0.02	0.51 ± 0.01	0.84 ± 0.00
AD vs. MCI vs. CN:	0.56 ± 0.02	0.32 ± 0.03	0.55 ± 0.02	0.78 ± 0.01
AD vs. CN vs. BV:	0.78 ± 0.03	0.55 ± 0.05	0.73 ± 0.02	0.87 ± 0.01
BV vs. CN:	0.88 ± 0.03	0.57 ± 0.03	0.90 ± 0.08	0.86 ± 0.02
AD vs. CN:	0.82 ± 0.04	0.61 ± 0.08	0.82 ± 0.05	0.82 ± 0.03
AD vs. BV:	0.93 ± 0.01	0.73 ± 0.04	0.85 ± 0.02	1.00 ± 0.00
**Independent test set (AIBL)**				
AD vs. MCI vs. CN:	0.53 ± 0.01	0.30 ± 0.03	0.69 ± 0.01	0.84 ± 0.01
AD vs. CN:	0.80 ± 0.01	0.59 ± 0.01	0.66 ± 0.02	0.94 ± 0.01

In a multi-class setup, micro averages are reported for the sensitivity and specificity metrics. CN, cognitively normal; AD, dementia due to Alzheimer's disease; MCI, mild cognitive impairment; BV, behavioral variant of frontotemporal dementia; MCC, Matthews correlation coefficient.

Upon comparing results from various settings of classification heads trained over a frozen feature extractor, we can observe a general trend, i.e., the binary classification for separating cognitively normal (CN) and Alzheimer's disease (AD) samples is a much simpler task than the 4-way multi-class classification of CN, mild cognitive impairment (MCI), AD and behavioral variant of frontotemporal dementia (BV) samples. This finding has often been reported in other studies in the field.

In the multi-class classification setting, the AD vs. MCI vs. CN model, often confuses MCI samples with CN or AD samples. This reflects the progressive nature of the Alzheimer's dementia, with MCI being intermediate stage between CN and AD. Interestingly, we found that the AD vs. MCI vs. CN vs. BV model is substantially better at separating BV samples from the other CN, MCI and AD samples, with the recall (=sensitivity) of the BV class being 0.89, compared to the average micro recall of the same model being 0.51. This finding points toward the model being sensitive to different underlying pathologies of different dementia diseases—frontotemporal dementia and AD. The same fact could also be corroborated from the high performance metrics of the binary AD vs. BV model. In Section 5.1 below, we discuss the achieved results and compare them with the state of the art.

### 4.2 Model interpretability

**Figure 3:**
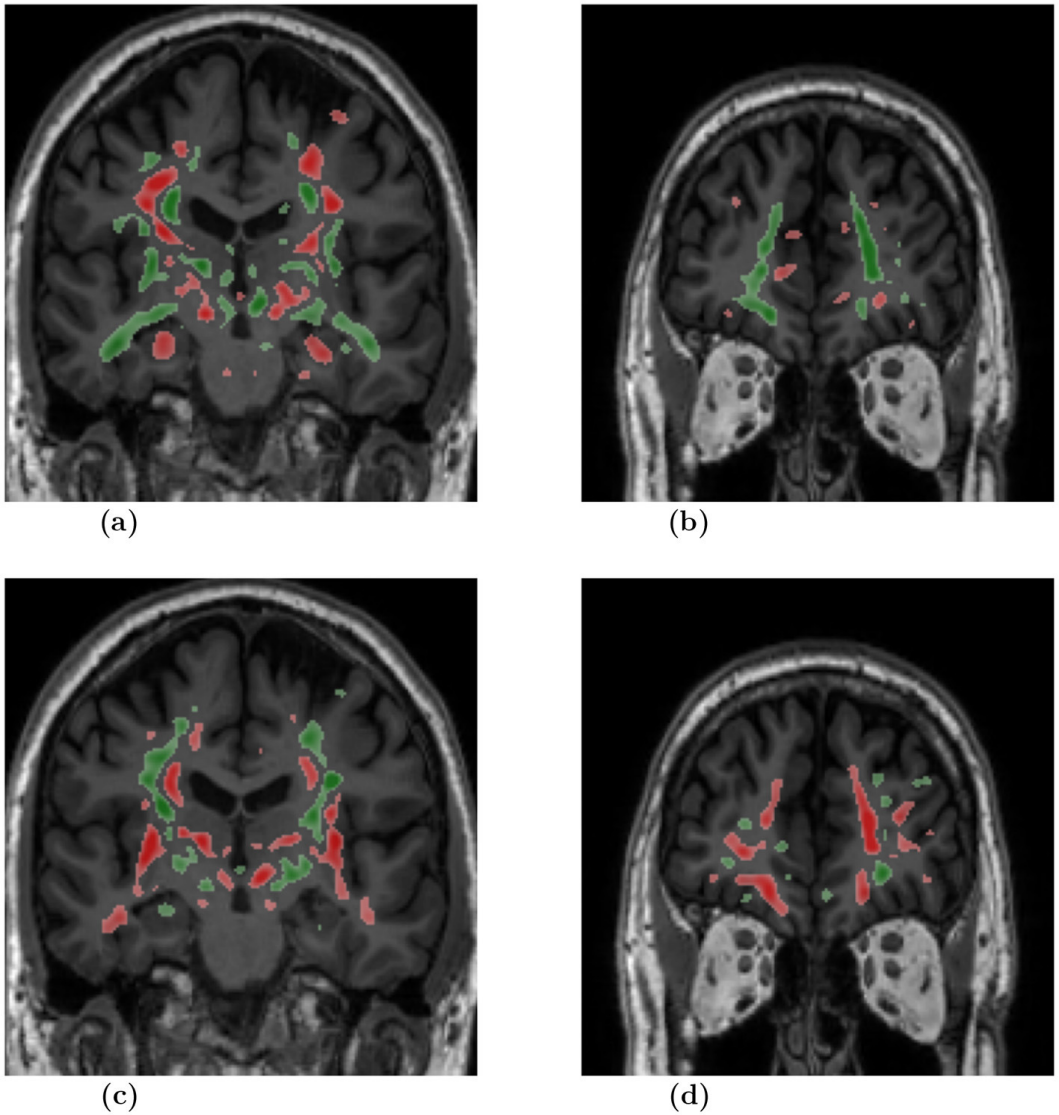
Mean attribution maps derived from the Integrated Gradients method for correctly identified AD and BV samples. Green and red color highlight pixel contributions to the model's prediction. Here, red highlights evidence for the respective disease classification and green indicates evidence against it. The attribution map overlay image was smoothed and thresholded to highlight relevant findings and improve visualization. AD, dementia due to Alzheimer's disease; BV, behavioral variant of frontotemporal dementia. **(A)** Slice: 0, Diagnosis: AD. **(B)** Slice: 60, Diagnosis: AD. **(C)** Slice: 0, Diagnosis: BV. **(D)** Slice: 60, Diagnosis: BV.

To highlight the input regions that were found to be useful by the SSL model, we used the Integrated Gradients (IG) attribution method. IG calculates the importance scores for the input regions for a specified prediction label. The IG importance scores were calculated for every sample of the test data set (from ADNI2/3 and FTLDNI), on which our multi-class model (AD vs. CN vs. BV) makes a correct classification. Figure 3 presents mean IG importance scores for the disease types AD and BV, visualized over the brain scan of a healthy sample chosen from the ADNI cohort. While making a prediction toward the diseased classes, the red regions in the image highlight input regions representing the evidence for the diseased class, while the green regions in the image highlight input regions representing the evidence against the diseased class. The mean importance scores were thresholded to visualize the most relevant findings.

## 5 Discussion

### 5.1 Feature learning

In our proposed SSL framework, we rely on signals that are derived from the data itself rather than on external classification target labels to train a feature extractor. We trained our SSL model while restricting input to a subset of 2D coronal slices. It should be noted that other SSL studies also avoided training 3D CNN with high input resolution and followed similar 2D approaches as our study (Couronné et al., 2021) or alternatively needed to drastically downscale the 3D images to a very low 64 × 64 × 64 resolution to reduce computing time (Ouyang et al., 2022; Fedorov et al., 2021).

Our AD vs. CN vs. BV multi-class model achieves a balanced accuracy of 78%. Certain fully supervised methods solve the same task, achieving performance metrics as—Ma et al. (2020) reports (simple) accuracy of 86.0% from a model comparable to ours and 88.3% from a model with multimodal information sources and generative data augmentation, and Hu et al. (2021b) reports (simple) accuracy of 66.8% on a larger diverse dataset, and 91.8% on a smaller cleaner dataset. While our BV vs. CN binary model achieves a balanced accuracy of 88.2%. For the same task Moguilner et al. (2023) reports (simple) accuracy of 80% and 95% on MRI scans with 1.5T and 3T strength, respectively.

There are other SSL studies that report AD vs. CN group separation results on the ADNI dataset. Dufumier et al. (2021) reported an AUC score around 0.96. Ouyang et al. (2022) achieved a balanced accuracy between 81.9% and 83.6%, pre and post model finetuning. Seyfioğlu et al. (2022) using a vision transformer reported a mean simple accuracy of 83.4%. While there also other SSL applications that reported sub-optimal results, Chen et al. (2023) reported a balanced accuracy between 68.23% and 77.5% depending on model architecture used, while Jiang and Miao (2022) reported a balanced accuracy between 73.1% and 74% depending on the pretext task used. For the same task reported in these studies, our model with a frozen feature extractor, achieves a balanced accuracy of 82% on ADNI dataset, which is competitive with metrics reported in other studies. And on a holdout independent test set (AIBL), our model achieves a balanced accuracy of 80%, which is only a two-percent drop from the cross-validation testing of the model, highlighting the robustness of the model. It should noted that many studies don't evaluate their models on a holdout independent test set, which makes it is difficult to access their generalizability.

In Table 3, we compare our model evaluation results with the state-of-the-art studies that also used AIBL as an independent test dataset. Here, we compare our SSL model with other models trained in a supervised manner. Qiu et al. (2020), reports manual expert rating scores, with a simple accuracy metric of 82.3%. This performance level is comparable to that of our SSL models, which achieved the simple accuracy measure of 89.9% on the AIBL independent test set. It should be noted that some papers did not report the *balanced accuracy* measure, thus, their “simple” accuracy results might be biased toward the majority class of cognitively normal people who comprise 80% in the AIBL dataset for the group comparison AD vs. sCN.

**Table 3:** Comparison of our proposed method with the state-of-the-art.

**Study training on the ADNI dataset**	**Method details**	**Balanced accuracy on the AIBL dataset**
Our method	SSL, 2D slice-level CNN	0.797 ± 0.009
Wen et al. (2020)	SL, 2D slice-level CNN	0.756 ± 0.015
Wen et al. (2020)	SL, 3D patch-level CNN	0.802 ± 0.016
Wen et al. (2020)	SL, 3D subject-level CNN	0.862 ± 0.016
Dyrba et al. (2021)	SL, 3D subject-level CNN	0.832 ± 0.030
		**Simple accuracy on the AIBL dataset**
Our method	SSL, 2D slice-level CNN	0.899 ± 0.003
Qiu et al. (2020)	SL, 3D patch-level CNN	0.870 ± 0.022
Han et al. (2022)	SL, 3D subject-level CNN	0.865
Han et al. (2022)	SL, 3D patch-level CNN	0.875
Qiu et al. (2020)	Expert Neurologists	0.823 ± 0.094

The results are provided for studies that used the AIBL dataset for independent evaluation and the group comparison AD vs. CN. In some studies balanced accuracy was not reported, “simple” accuracy is provided instead, which might be biased toward the majority class (=CN). AD, dementia due to Alzheimer's disease; CN, cognitively normal; SSL, Self-supervised learning; SL, Supervised learning; CNN, Convolutional neural network.

With regard to our achieved level of performance, we can conclude that the ConvNeXt model trained under a SSL paradigm learns generalizable features for the subsequent downstream classification tasks without requiring data sampling techniques or sophisticated data augmentations, and consequently achieving competitive results in comparison to other supervised approaches. The reported results show that our model learned meaningful feature representations in a self-supervised manner, which can be used successfully to separate different stages and types of dementia.

### 5.2 Neural network interpretability

We chose the SSL paradigm to extract more generalizable image features independently of a downstream task. However, the SSL paradigm also allows the backbone model to learn features of the brain that may correlate with a specific neurodegenerative disorder. We applied the Integrated Gradients (IG) method to interpret the models and provide insights into the significance of input regions for the predictions. The IG importance scores were calculated for samples from the test dataset for which our AD vs. CN vs. BV multi-class model makes correct classifications. Figure 3 illustrates the mean IG importance scores for classifying samples into the AD or BV group. In Figures 3A, B, we see the hippocampus region highlighted in red for AD classification. Temporal lobe atrophy, specifically hippocampus atrophy, is a hallmark sign of Alzheimer's disease. In Figures 3C, D, we see the insula and frontal lobe regions being highlighted in red. Insular atrophy is associated with the behavioral variant of frontotemporal dementia (Moguilner et al., 2023; Seeley, 2010; Luo et al., 2020; Mandelli et al., 2016). It is of great interest to see the IG maps separately highlighting regions, which in the literature are often associated with AD and BV pathology.

Furthermore, to our knowledge, only one previous study, Dadsetan et al. (2022), has systematically compared different pretext methods for training SSL models for AD progression prediction, while also employing an XAI method, i.e., GradCAM, to generate relevance maps to evaluate the learned features. However, the reported relevance maps were particularly diffuse and widespread, offering limited interpretability. In addition, as an ablation study, we investigated different XAI methods beyond IG, but the results of these experiments also produced diffuse, spiky and unspecific relevance maps. This highlights that the application of XAI methods to SSL methods remains an open area of research.

Notably, our model successfully learned to not consider tissue outside of the brain or regions outside of the skull. However, the derived attributions provide a rather general indication of important input regions throughout the brain, including primarily gray matter and white matter tissue. Few studies have pointed out the complex nature of IG importance scores that highlight multiple image features, both for and against a class instance, making their comprehension non-trivial (Adebayo et al., 2018; Kakogeorgiou and Karantzalos, 2021; Hiller et al., 2025).

### 5.3 Limitations and future work

Our study uses only a subset of coronal slices to make sample-level classifications. We acknowledge that the selection of the full slice set along the coronal axis or selection of the full 3D MRI data could have a positive effect on classification performance; however, the main goal of the study was to investigate the application of SSL and to compare it with traditional supervised approaches; thus only a subset of slices along the coronal axis was chosen as input. Learning a 3D CNN is a computationally expensive problem for self-supervised learning, as it relies on (a) very large data corpus, (b) data augmentation algorithms which are markedly more computationally expensive in 3D due to the cubic time-complexity of the algorithms, and (c) many learning iterations as training typically converges much slower than in supervised learning. More specifically, training our models for 1,000 epochs on a single NVIDIA Quadro RTX 6000 GPU took on average 27 h. In the future, to train better feature extractors, we will incorporate more spatial neuroanatomical information, by combining three CNNs, i.e., one trained along each orthogonal planes—axial, coronal, and sagittal, and hence learning feature representations for the full 3D MRI data, as was proposed for supervised models (Qiao et al., 2021). Alternatively, a vision transformer model could also be explored to efficiently process smaller 3D patches of the brain (Qiu et al., 2020; Wen et al., 2020; Han et al., 2022; Wolf et al., 2023).

With regard to neural network interpretability and feature attribution, a comprehensive analysis of the salient features and feature attribution methods lies outside the scope of our current work. Although it remains to be seen whether the somewhat dispersed attribution maps we see in the current study are due to a difference in the training paradigm, i.e., SSL vs. supervised learning. To the best of our knowledge, no systematic efforts have been undertaken to compare the effects of training paradigm and attribution methods in highlighting disease-specific brain structures known in the clinical literature for different types of dementia. Additional experiments are required to holistically understand our SSL model and the informative importance of the generated maps. In our future work, we will explore other methods for feature attribution and methods to summarize attributions per brain region to assess if specific disease patterns emerge.

We also intend to include additional datasets in our future studies to learn more robust models. Specifically, we intend to add FTLD data cohorts.

### 5.4 Conclusion

We presented an architecture for the identification of neurodegenerative diseases from MRI data, consisting of a feature extractor and a classification head. The feature extractor used the ConvNeXt architecture as a backbone, which was trained under a self-supervised learning paradigm with nearest-neighbor contrastive learning (NNCLR) loss on brain MRI scans. The feature extractor model was used for subsequent downstream tasks by training only an additional single-layer neural network component which performs the classification. From our experiments, we show that CNN models trained under SSL paradigm have comparable performance to state-of-the-art CNN models trained in a supervised manner. With this presented approach, we provide a practical application of self-supervised learning on MRI data, as well as also demonstrate the application of attribution mapping methods for such systems to improve interpretability of the model's decision.

## Data Availability

Publicly available datasets were analyzed in this study. This data can be found here: Alzheimer's Disease Neuroimaging Initiative (ADNI) (http://adni.loni.usc.edu/data-samples/access-data), Australian Imaging Biomarkers and Lifestyle flagship study of aging (AIBL) (https://aibl.csiro.au), and Frontotemporal Lobar Degeneration Neuroimaging Initiative (FTLDNI) (https://memory.ucsf.edu/research-trials/research/allftd). Our source code for data processing, model training and evaluation, and creating attribution maps will be made publicly available at: (https://github.com/VadymV/clinic-net).

## Footnotes

^1^ADNI: https://adni.loni.usc.edu/.

^2^AIBL: https://aibl.csiro.au/.

^3^FTLDNI: https://memory.ucsf.edu/research-trials/research/allftd.

## For the ADNI, AIBL, FTLDNI study groups

A complete listing of ADNI investigators can be found at https://adni.loni.usc.edu/wp-content/uploads/how_to_apply/ADNI_Acknowledgement_List.pdf. AIBL researchers are listed at https://www.aibl.csiro.au.
