# Supplementary material for: Contrastive self-supervised learning for neurodegenerative disorder classification
Source: Front Neuroinform. 2025 Feb 17;19:1527582. doi: 10.3389/fninf.2025.1527582 (PMC11873101; doi:10.3389/fninf.2025.1527582)
Supplement: Supplementary file 1 [file Data_Sheet_1.pdf]

Supplementary For ‘Contrastive Self-supervised Learning for Neurodegenerative Disorder Classification’

Training Feature Extractor

The ConvNeXt-tiny feature extractor was trained with the Nearest-Neighbor Contrastive Learning (NNCLR) loss, where data augmentation procedures were used on brain MRI scans for creating the positive pairs. Figure A.1(a) shows the training curve over one thousand epochs. During the first one hundred epochs, the contrastive loss decreases rapidly, while it slowly begins to saturate in the remaining epochs. This loss saturation suggests that the model has converged.

We experimented with effect of data source, pre-training, and weights being kept frozen or unfrozen. We loaded the ConvNeXt model weights pretrained on the ImageNet dataset. This model variant was trained together with the classification head, i.e., with unfrozen backbone model where the model weights were allowed to change. This setup was compared against a ConvNeXt model variant trained under the NNCLR SSL paradigm and the model weights kept frozen during the classification head training.

Figure A.1 (b) reports the training losses observed in these two scenarios, upon training the classification head for 100 epochs. The training loss curves suggests that the ConvNeXt backbone model when trained with the brain MRI scans (the medical datasets) learns more generalizable image features, and with frozen model weights while training the classification head, the whole model converges much faster. Hence, in our study this setup for training the backbone model was utilized, unless stated otherwise.

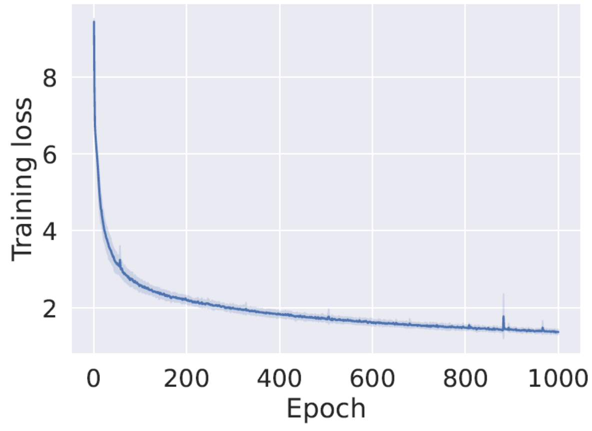

(a) Self-supervised ConvNeXt training

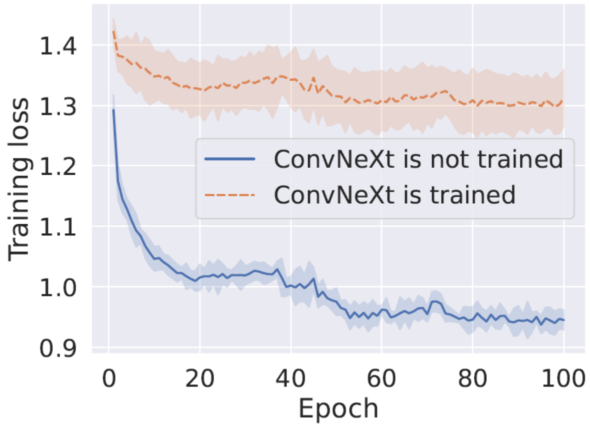

(b) Classification component training

Fig A.1 Loss curves of the models trained for three learning trials. Shaded areas denote 1 standard deviation. (a) Self-supervised training of the ConvNeXt feature extractor network via the NNCLR method. (b) Comparison of the classification head training following two strategies: with a ConvNeXt feature encoder backbone that was previously trained on the MRI data using the self-supervised NNCLR method and was not trained further during classifier training (blue solid line); and a ConvNeXt feature encoder backbone with the initial ImageNet model weights loaded and which was trained together with the classification head (supervised training, red dashed line).
